# Is “capacitive coupling” purely excitatory in the cardiac tissue?

**DOI:** 10.3389/fphys.2014.00077

**Published:** 2014-03-25

**Authors:** Alireza Akbari, Keivan Moradi, Fatemeh Hadaeghi, Shahriar Gharibzadeh, Zahra Emkanjoo

**Affiliations:** ^1^Department of Cardiac Electrophysiology, Cardiac Electrophysiology Research Center, Rajaie Cardiovascular Medical and Research Center, Iran University of Medical SciencesTehran, Iran; ^2^Department of Bioelectricity, Neural and Cognitive Sciences Laboratory, Biomedical Engineering Faculty, Amirkabir University of TechnologyTehran, Iran

**Keywords:** cardiac ephapse, intercalated disk, action potential propagation, conduction velocity, gap junction ablation

Cardiac muscle is a syncytial tissue of electrically coupled cells in which excitation of one cardiomyocyte spreads to all cardiomyocytes. Mechanisms of cell coupling in myocardial tissue can be categorized in two main groups: mechanisms that connect the intracellular parts of myocytes together (i.e., gap junctions), and mechanisms that act through changes in the small extracellular space of intercalated disks (ICD). The latter mechanisms are electric field coupling, ephaptic coupling, K accumulation, and capacitive coupling (Sperelakis and McConnell, 2002). ICD cleft has a limited space and therefore a small ionic current can change both its electric and diffusion potentials via the change in the ionic concentration. The potassium accumulation, for instance, can change the reversal potential of potassium channels toward more positive values, and as a results of which the potassium current can depolarize the cells. When ionic currents change the electric potential of cleft we have ephaptic or electrical coupling, and when the capacitive current of membrane changes the electric potential we have capacitive coupling.

In electrical coupling through gap junctions, both experimental and modeling studies show that the reduction of propagation velocity is proportional to the reduction of gap junction density or conductance. In the case of coupling through ICD field potential, since the experimental data are based on the effect of these mechanisms on conduction velocity (not a direct measurement of the field potential), there are significant discrepancies between different experiments, and between experiments and simulation studies (Rhett et al., 2013). One explanation of these differences is to consider a mixture of excitatory and inhibitory effects for each of these mechanisms and suppose that each experiment grasp one feature of these mechanisms based on the experimental condition. Such a mixed behavior was proposed for the ephaptic coupling in a previous study, where the voltage gated sodium channels of the ICD negatively impacted the conduction velocity unless more than 10% of the gap junctions remained active. Further reduction of gap junction density was reversed their impact (Kucera et al., 2002).

**Figure 1:**
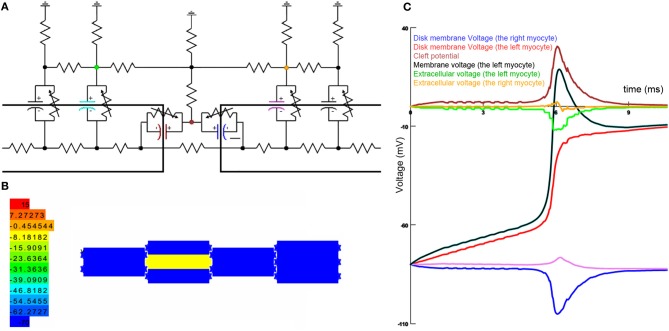
**Capacitive coupling simulation**. **(A)** The equivalent circuit of intercalated disk. **(B)** Cell arrangement in the final model. Colors show the membrane potential in all cells. **(C)** Membrane potential and extracellular voltage in different locations of the intercalated disk. Colors show the related parts in the equivalent circuit.

In the case of capacitive coupling, also, we suggest that a mixture of excitatory and inhibitory effects take place; so that depending on the compartment on which the membrane potential is measured, different effects may be observed. In capacitive coupling, we have two capacitors that share a voltage node, but each end of these capacitors are connected to different intracellular voltage nodes that provide the main polarity of the capacitors. Since these capacitors have a different polarity (Figure 1A), discharge (depolarization) of one of them by action potential generation would charge (hyperpolarize) the other one. Still, these capacitors in series can pass the current, which would increase the potential in the next intracellular voltage node. The hyperpolarization of ICD membrane was reported in a previous modeling study, but due to the complex nature of their model it was impossible to dissociate the electrical field coupling from the capacitive coupling (Ge and Sperelakis, 1992).

We have simulated ICD in the NEURON simulation environment (Hines and Carnevale, 1997). In our model, an equivalent circuit of the ICD connects 10 myocytes together in the ICD (Figure 1B). Our model could potentially simulate ion channels in the cleft and gap junction. Capacitive coupling is the property of membranes lacking ion channels. Therefore, in the Figure 1C, we show results of this model where there is no gap junction and ion channels in the ICD. The results of this model clearly show that the capacitive coupling hyperpolarizes the neighboring membrane in the ICD and depolarizes the adjacent myocyte membrane to some extent. Overall, based on the abovementioned simulation we hypothesize that the capacitive coupling has mixed excitatory and inhibitory effects. Therefore, we suggest that action potential propagation models should be revised based on this finding.
